# Designing a large language model for chemists

**DOI:** 10.1016/j.patter.2025.101264

**Published:** 2025-05-09

**Authors:** Xiaoyi Chen, Haixu Tang

**Affiliations:** 1 Luddy School of Informatics, Computing, and Engineering, Indiana University Bloomington, Bloomington, IN, USA

## Abstract

In a recent issue of *Cell Reports Physical Science*, Zhao et al. introduced ChemDFM, a foundational large language model designed specifically for chemistry. The model bridges the gap between general-purpose language models and specialized chemical knowledge, including the integration of multimodal capabilities for spectroscopic data interpretation, improved numerical reasoning, and connectivity with chemical tools and databases to enhance practical research applications. This approach demonstrates how domain adaptation can transform AI tools into collaborative research partners for scientific discovery.

## Main text

The application of artificial intelligence (AI) in chemistry has witnessed remarkable progress, as evidenced by recent studies highlighting its expanding influence.^1^^,^^2^ Building upon the transformative advancements of language models like BERT^3^ and GPT,^4^ researchers have successfully adapted these pre-trained models for chemical applications.^5^^,^^6^ However, a critical limitation persists: the majority of current chemical AI systems are highly specialized, engineered for isolated tasks with specific datasets. This narrow focus results in a fragmented ecosystem where models exhibit proficiency only within constrained domains, hindering their adaptability to related chemical challenges.

The chemistry community is increasingly advocating for adaptable AI systems that can seamlessly address diverse challenges across chemical domains and facilitate natural interactions with researchers. Recent breakthroughs in large language models (LLMs), such as GPT-4^7^ and LLaMA,^8^ demonstrate promising abilities in reasoning, knowledge synthesis, and task generalization. However, these general-purpose LLMs encounter substantial obstacles when applied to chemistry. A critical deficiency lies in their lack of domain-specific understanding, particularly concerning specialized chemical notations. For example, the notation “CO” signifies carbon monoxide to a chemist, not the state of Colorado, while “Co” denotes cobalt, not a company. General LLMs often misinterpret these fundamental chemical representations, severely limiting their utility in research settings.

A truly effective chemical AI system would bridge this gap, integrating the reasoning power of LLMs with deep chemical knowledge. Such a system could potentially serve as a collaborative research partner, understanding both natural language instructions and chemical representations—a significant step toward realizing chemical artificial general intelligence. In a recent issue of *Cell Reports Physical Science*, Zhao et al. present ChemDFM,^9^ a pioneering effort toward this vision.

ChemDFM addresses this critical need through a two-stage specialization process: domain pre-training followed by instruction tuning. Leveraging the open-source LLaMA-13B model, the researchers conducted domain pre-training using an extensive corpus of chemical literature, which contains 34 billion tokens extracted from over 3.8 million papers and 1,400 textbooks. Subsequently, they refined the model through instruction tuning with 2.7 million chemistry-focused instructions derived from chemical databases. This approach enables ChemDFM to preserve the reasoning capabilities of general LLMs while gaining deep chemical expertise.

The authors evaluated ChemDFM against LLMs including GPT-4,^7^ LLaMA-2,^8^ and Galactica^10^ on various chemical tasks. ChemDFM demonstrated superior performance compared with other open-source LLMs and even outperformed GPT-4 on many chemistry-specific challenges, which is particularly noteworthy given that ChemDFM is based on an LLM with 13 billion parameters, whereas GPT-4 is vastly larger. For instance, for the task of text-based molecule design, ChemDFM outperformed both general-purpose and specialized LLMs. For the task of molecular property prediction, although it is still behind task-specific specialist models (as expected for a generalist model), ChemDFM significantly outperformed other LLMs, including GPT-4. The study identified limitations of ChemDFM as well, particularly for the tasks of numerical computation and reaction yield prediction.

Beyond benchmarks, the paper demonstrates ChemDFM’s potential as a research assistant through realistic scenarios in literature reading and experimental design. One example shows the model assisting a researcher with selective oxidation, showcasing its ability to understand chemical questions, correct errors, and provide detailed recommendations while maintaining natural dialogue. This capability represents a significant advance in human-AI collaboration for chemistry research.

One of ChemDFM’s most significant contributions is its ability to connect natural language and specialized chemical notation. Chemistry has developed its specific languages, such as SMILES notation, IUPAC names, and molecular formulas, which are efficient for representing complex structures of chemical compounds but challenge general-purpose LLMs. The instruction tuning phase directly addressed this representational gap by incorporating tasks such as molecular notation alignment, effectively training the model to seamlessly translate between diverse molecular representations.

ChemDFM’s success has several important implications. First, it demonstrates an effective approach to domain adaptation for LLMs, providing a template applicable to other scientific disciplines. The two-stage specialization process offers a systematic method for infusing domain expertise into general-purpose LLMs. Second, it highlights the importance of careful data curation. The selection of chemical literature, textbooks, and task-specific training data proved crucial to the model’s performance, underscoring the value of domain expertise in AI development. Finally, ChemDFM signals a paradigm shift, heralding AI systems that function as integral collaborators in scientific research rather than mere task executors. By facilitating natural dialogue and leveraging deep chemical expertise, it promises to transform human-AI collaboration and accelerate scientific research.

To further advance this work, researchers might consider several promising enhancements. Multimodal integration would empower ChemDFM to analyze spectroscopic and microscopic data, which is critical for experimental insights. Improved numerical reasoning capabilities would refine predictions of reaction yields and other quantitative parameters. Direct integration with chemical tools and databases would extend the model’s practical utility in active research environments.

Despite promising initial results, the development of truly capable chemical language models still poses significant hurdles. A primary obstacle lies in the quality and comprehensiveness of training data. While ChemDFM incorporates an impressive corpus, the rapid pace of chemical research makes achieving comprehensive coverage of the latest findings exceedingly difficult. Continuously ensuring that models accurately reflect current research, without introducing hallucinations or biases present in the literature, demands ongoing and rigorous attention. For specialized domains underrepresented in training data, such as experimental troubleshooting, supplementary techniques like retrieval-augmented generation offer a potential solution by dynamically integrating external knowledge sources.

Another substantial challenge stems from the considerable computational resources required for both training and deploying advanced chemical language models. The pursuit of more capable models often requires greater computational power, potentially limiting accessibility. Developing more efficient model architectures and training methodologies is crucial for democratizing access to these powerful tools.

Finally, the effective evaluation of chemical language models presents unique complexities. The field requires more comprehensive benchmarks that move beyond assessing isolated tasks to evaluate integrated research capabilities—specifically, how well models can reason through complex, multistep problems. This requires close collaboration between AI researchers and practicing chemists to establish meaningful evaluation criteria, design realistic test cases that mirror actual research scenarios, and provide expert assessment of model outputs.

By open-sourcing ChemDFM, the authors have invited broader participation in addressing these challenges. This collaborative approach promises to accelerate progress toward AI systems that serve as valuable partners in chemical research and discovery.

## Declaration of interests

The authors declare no competing interests.
